# Bis(aminothiolato)nickel nanosheet as a redox switch for conductivity and an electrocatalyst for the hydrogen evolution reaction†
†Electronic supplementary information (ESI) available. See DOI: 10.1039/c7sc02688a


**DOI:** 10.1039/c7sc02688a

**Published:** 2017-10-03

**Authors:** Xinsen Sun, Kuo-Hui Wu, Ryota Sakamoto, Tetsuro Kusamoto, Hiroaki Maeda, Xiaojuan Ni, Wei Jiang, Feng Liu, Sono Sasaki, Hiroyasu Masunaga, Hiroshi Nishihara

**Affiliations:** a Department of Chemistry , School of Science , The University of Tokyo , 7-3-1 Hongo, Bunkyo-ku , Tokyo , 113-0033 , Japan . Email: nisihara@chem.s.u-tokyo.ac.jp; b Department of Materials Science and Engineering , University of Utah , Salt Lake City , UT 84112 , USA; c Faculty of Fibre Science and Engineering , Kyoto Institute of Technology , Matsugasaki Hashikami-cho 1, Sakyo-ku , Kyoto , 606-8585 , Japan; d RIKEN SPring-8 Centre , Kouto 1-1-1, Sayo-cho, Sayo-gun , Hyogo , 679-5148 , Japan; e Japan Synchrotron Radiation Research Institute (JASRI)/SPring-8 , 1-1-1 Kouto, Sayo-cho, Sayo-gun , Hyogo , 679-5198 , Japan

## Abstract

A π-conjugated coordination nanosheet comprising bis(aminothiolato)nickel (**NiAT**) moieties was synthesized by the reaction of Ni(acac)_2_ with 1,3,5-triaminobenzene-2,4,6-trithiol at liquid–liquid and gas–liquid interfaces. The sheet thickness could be controlled down to a single layer (0.6 nm). Selected area electron diffraction and grazing incidence X-ray diffraction analyses indicated the formation of a flat crystalline sheet with a kagome lattice stacked in a staggered alignment. **NiAT** was reversibly interconverted to a bis(iminothiolato)nickel (**NiIT**) nanosheet by the chemical 2H^+^–2e^–^ reaction, which was accompanied by a drastic change in electrical conductivity from 3 × 10^–6^ to 1 × 10^–1^ S cm^–1^. This change in conductivity was explained by the difference in band structures between **NiAT** and **NiIT**. **NiAT** acted as an efficient electrocatalyst for the hydrogen evolution reaction, showing strong acid durability and an onset overpotential of –0.15 V.

## Abstract 2

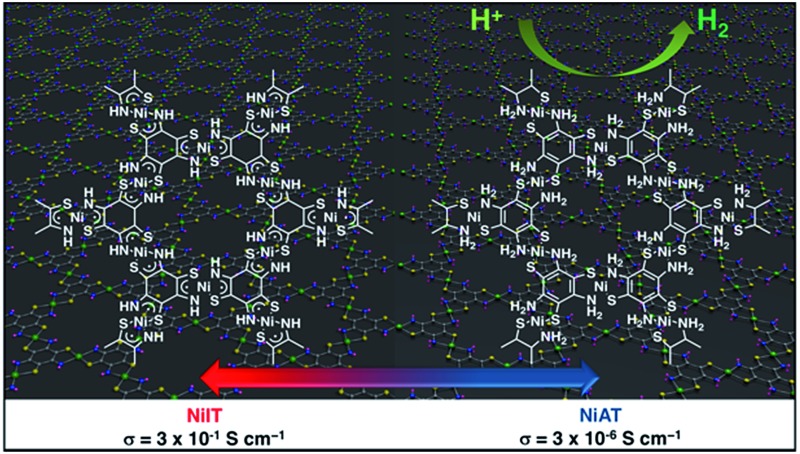
Precisely tuned functional coordination nanosheet exhibits competent catalytic activity for HER, accompany with drastic electronic property switching by redox treatment.

## Introduction

Two-dimensional materials, such as graphene,^1^,^2^ metal oxides,^3^,^4^ chalcogenides,^5^–^7^ hydroxides,^8^–^10^ and covalent organic frameworks,^11^–^14^ have attracted much attention because of their unique properties. These materials have various potential applications, including transistors,^15^–^17^ sensors,^18^,^19^ luminescent devices,^20^–^22^ thermal interface materials,^23^,^24^ and piezo elements.^25^ Another type of intriguing 2D material is coordination nanosheets (CONASHs),^26^,^27^ which consist of metal complexes and can exhibit various unique physical and chemical features. They are easy to make by convenient bottom-up syntheses, because many coordination reactions proceed in solution under ambient conditions. The oil–water (liquid–liquid) interface can be used as the coordination reaction field for metal ions and ligands if they are soluble only in different phases. Initially, the characterization and structural analysis of CONASHs were the main focuses of research.^28^–^41^ Recently, various functionalities of CONASHs have been demonstrated.^42^–^47^

2D materials based on the bis(dithiolato)metal complex motif and its analogues comprise important series in CONASHs. Their square-planar geometry is ideal for constructing 2D frameworks. Our first report^48^ has stimulated their variations, in combination with metal ions (Ni^II^, Pd^II^, and Cu^I^) with aromatic organic ligands (triphenylenehexaol,^49^,^50^ benzenehexathiol,^48^,^51^–^53^ triphenylenehexaamine,^50^,^54^–^57^ triphenylenehexathiol,^58^,^59^ and benzenehexaamine^60^). They exhibit high electrical conductivity due to strong charge delocalization in the plane,^53^–^57^,^61^ some of them been theoretically predicted to be 2D topological insulators.^62^ Moreover, these CONASHs can also act as electrocatalysts: for example, the hydrogen evolution reaction (HER) was catalysed by the bis(dithiolato)metal type of CONASHs.^58^,^63^,^64^

**Fig. 1:**
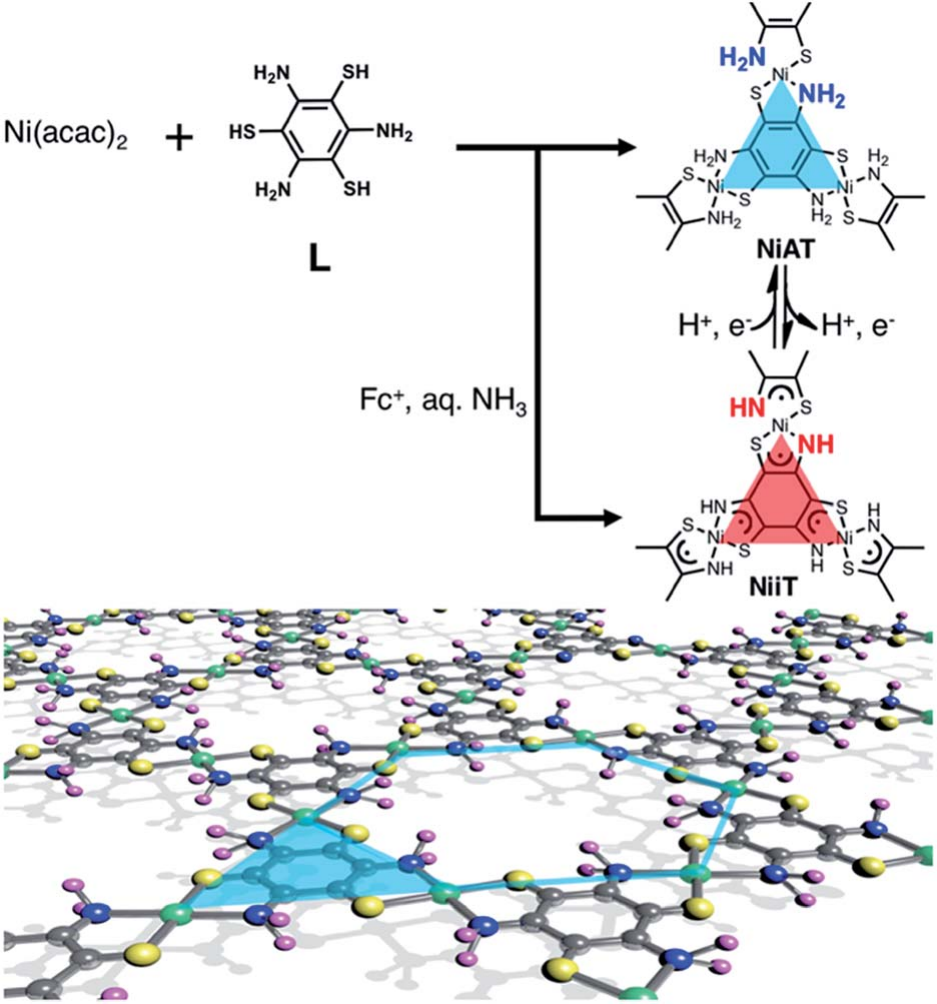
Schematics and chemical structures of **NiAT** and **NiIT**. Grey: C; yellow: S; blue: N; purple: H; green: Ni.

Herein, we add a new aspect in the bis(dithiolato)metal type of CONASHs, by creating a crystalline π-conjugated CONASH comprising bis(aminothiolato)nickel (**NiAT**, Fig. 1) synthesized by the interfacial reaction of 1,3,5-triaminobenzene-2,4,6-trithiol (**L**) in water and bis(2,4-pentanedionato)nickel(ii) (Ni(acac)_2_) in CH_2_Cl_2_. We recently reported the synthesis of a bis(iminothiolato)nickel (**NiIT**) nanosheet (Fig. 1),^65^ although the reaction conditions were different. **NiAT** has composition and structure similar to those of **NiIT**, but different coordination modes with each other. The similarity allows **NiAT** and **NiIT** to be interconvertible reversibly *via* the chemical 2H^+^–2e^–^ reaction per nickel complex unit, while the difference induces a drastic change in electrical conductivity. This change is explained by the obvious difference in band structures between **NiAT** and **NiIT**. We also find that **NiAT** exhibited high, durable catalytic activity for the HER.

## Results and discussion

**Fig. 2:**
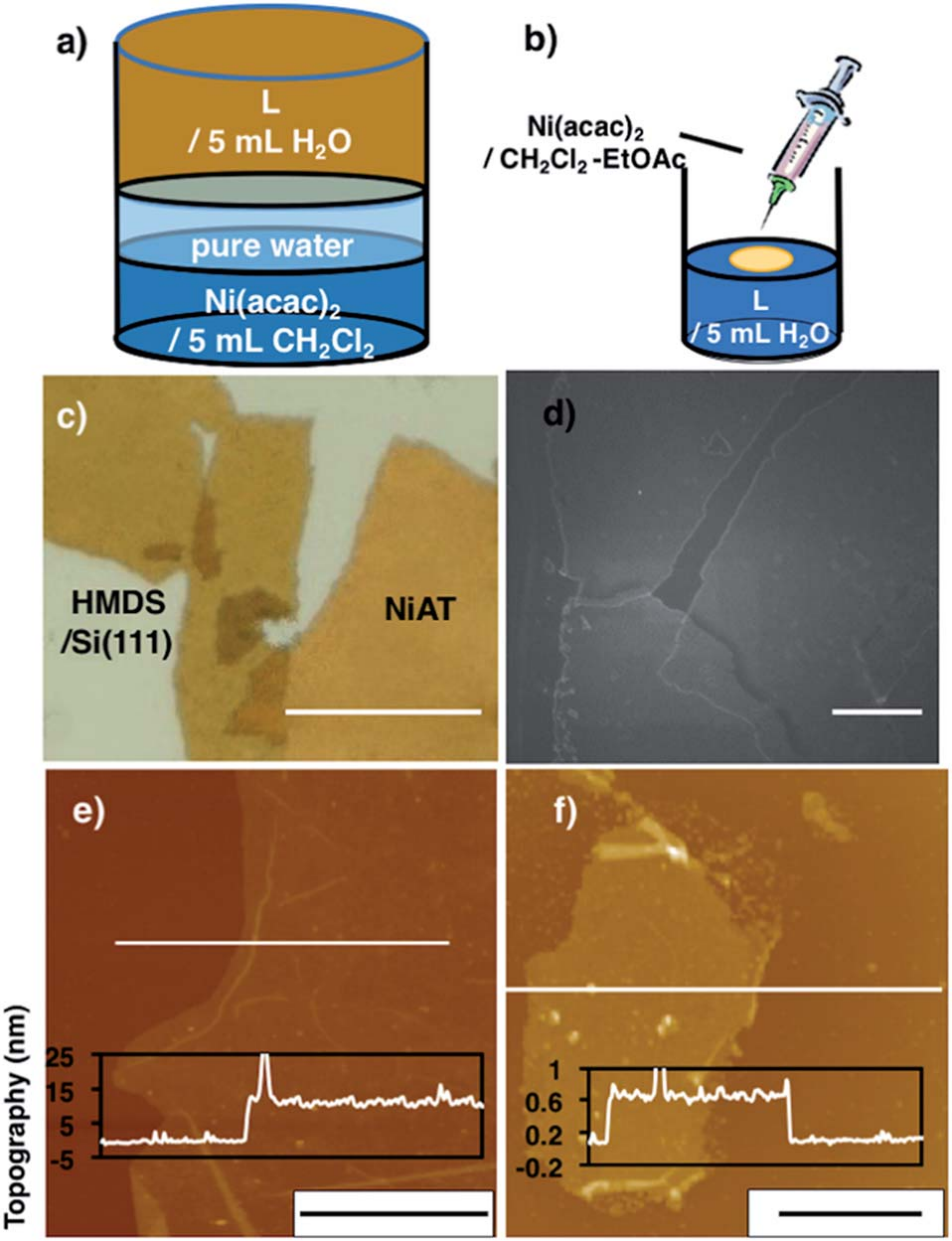
(a) Schematics of the liquid–liquid interfacial synthesis of multi-layer **NiAT**. (b) Schematics of the gas–liquid interfacial synthesis of single-layer **NiAT**. (c) Optical microscope images of multi-layer **NiAT** on HMDS/Si(111). Scale bars represent 50 μm. (d) FE-SEM images on HMDS/Si(111) of multi-layer **NiAT**. Scale bars represent 50 nm. (e) AFM image on HMDS/Si(111) and its cross-section analysis along the white line of the multi-layer. Scale bars represent 10 μm. (f) AFM image on HMDS/Si(111) and its cross-section analysis along the magenta line along the white line of the single-layer. Scale bars represent 2 μm. AFM images of (e) and (f) including height distribution bar with roughness analysis along magenta lines (Fig. S1†).

Typical fabrication processes of multi-layered and single-layered **NiAT***via* an interfacial reaction are shown in Fig. 2a and b, respectively. The multi-layered sheets were obtained at the liquid–liquid interface using an aqueous solution of **L** (1 μM) and a CH_2_Cl_2_ solution of Ni(acac)_2_ (0.5 mM). The bilayer solution was stood at room temperature under argon for 8 days, and a light brown-coloured thin film formed at the liquid/liquid interface. The film was cleaned by repeatedly replacing each solution with pure solvent. The film was vertically transferred onto a flat silicon(111) crystal plate chemically modified with 1,1,1,3,3,3-hexamethyldisilazane [HMDS/Si(111)].

Optical microscopy revealed yellow colored film with lateral lengths of more than 50 μm (Fig. 1c) and field-emission scanning electron microscopy (FE-SEM) of the film revealed flat and layered film morphologies (Fig. 1d). Atomic force microscopy (AFM) showed a flat, thin film façade with a thickness of 12 nm (Fig. 2e), which contained *ca.* 30 layers, based on the thickness of a single layer on [HMDS/Si(111)] of 0.6 nm and the interlayer distance of **NiAT** of 0.42 nm. Wrinkles and folded sections were observed close to the edge of sheet, suggesting a uniform, pliable sheet material.

Single-layer **NiAT** was fabricated by a gas–liquid interfacial reaction (Fig. 2b). A tiny amount of Ni(acac)_2_ in CH_2_Cl_2_–EtOAc (10 : 1 v/v) was spread onto an aqueous solution of **L** (35 μM) under argon. Composition of highly extensible ethyl acetate let the organic solution spread on water surface immediately, leading to rapid evaporation of solvent. After evaporation of the organic solvents, the coordination reaction proceeded at the gas–liquid interface to form an ultrathin film, which was transferred onto HMDS/Si(111). Fig. 2f shows that the film was 0.6 nm thick, corresponding to a single layer.

**Fig. 3:**
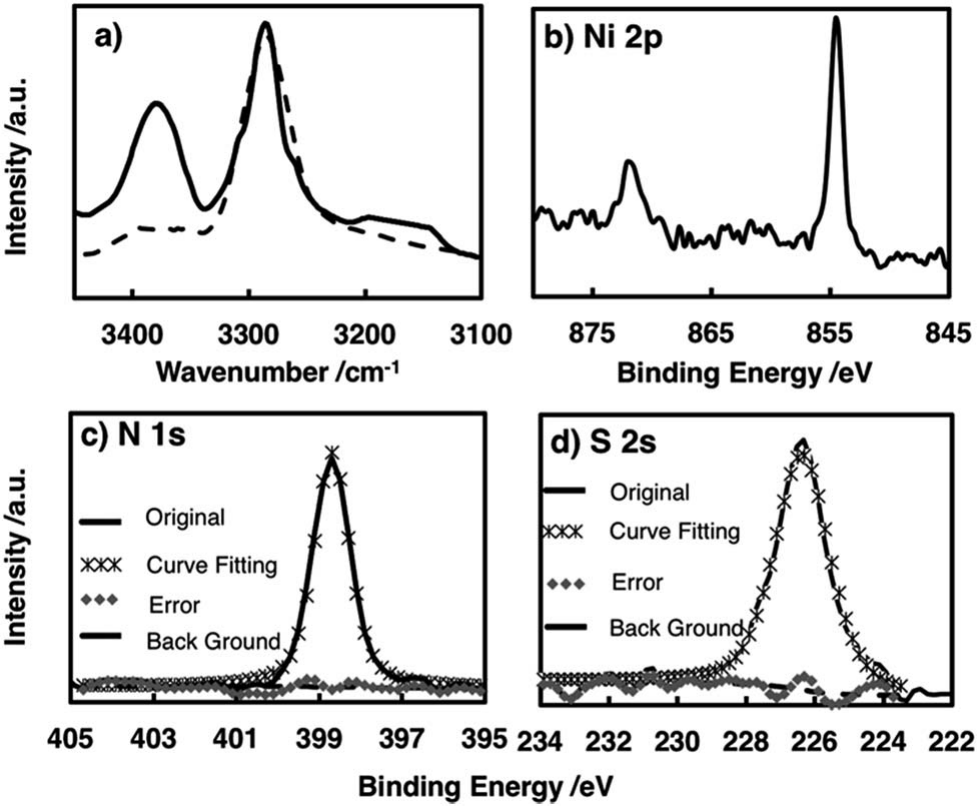
(a) IR spectra in the N–H stretching vibration region of **NiAT** (solid line) and **NiIT** (dashed line). Narrow-scan XPS focusing on (b) the Ni 2p region, (c) the N 1s region, and (d) the S 2s region.

The chemical structure of the metal complex unit in the film was identified by attenuated total reflectance (ATR)-IR spectroscopy. The multi-layered film showed no S–H stretching vibration signal around 2500 cm^–1^, whereas this signal was observed for **L** (Fig. S2†), indicating that all the thiol groups of **L** participated in coordinating to the Ni centre.^48^ The amino N–H showed two bands ascribed to antisymmetric and symmetric stretching vibrations, whereas the imino N–H exhibited only one N–H stretching vibration band. The spectrum of the film contained two peaks at 3270 and 3380 cm^–1^, confirming the presence of the –NH_2_–Ni motif in **NiAT**. The previously reported **NiIT** film showed a single peak at 3270 cm^–1^ (Fig. 3a).

X-ray photoelectron spectroscopy (XPS) of **NiAT** detected N, S, and Ni in a ratio of 1.98 : 2.07 : 1 which was consistent with the ideal ratio of 2 : 2 : 1 and indicated the quantitative formation of nickel complex units in the nanosheets (Fig. 3b–d). For reference, XPS spectra of **L**, the **NiIT** nanosheet, and the neutral mononuclear complexes, bis(1-aminobenzene-2-thiolato)nickel(ii) (**NiAT-M**) and bis(1-iminobenzene-2-thiolato)nickel(ii) (**NiIT-M**),^66^ were also obtained (Fig. S3–S6 and Table S1†). Two Ni 2p peaks at binding energies of 855 and 873 eV for **NiAT** were assigned to Ni 2p_3/2_ and Ni 2p_1/2_, respectively (Fig. 3b). The N 1s peak for **NiAT** was observed at 398.7 eV (Fig. 3c), slightly lower than that of **NiIT** at 399.9 eV (Fig. S6 and Table S1†), which was similar to the comparison of **NiAT-M** and **NiIT-M**. Only one S 2s peak appeared at 226.5 eV for **NiAT** (Fig. 3d), indicating that the nickel complex unit was in a neutral oxidation state. In conclusion, the **NiAT** film contained only one type of bis(aminothiolato)nickel moiety.

**Fig. 4:**
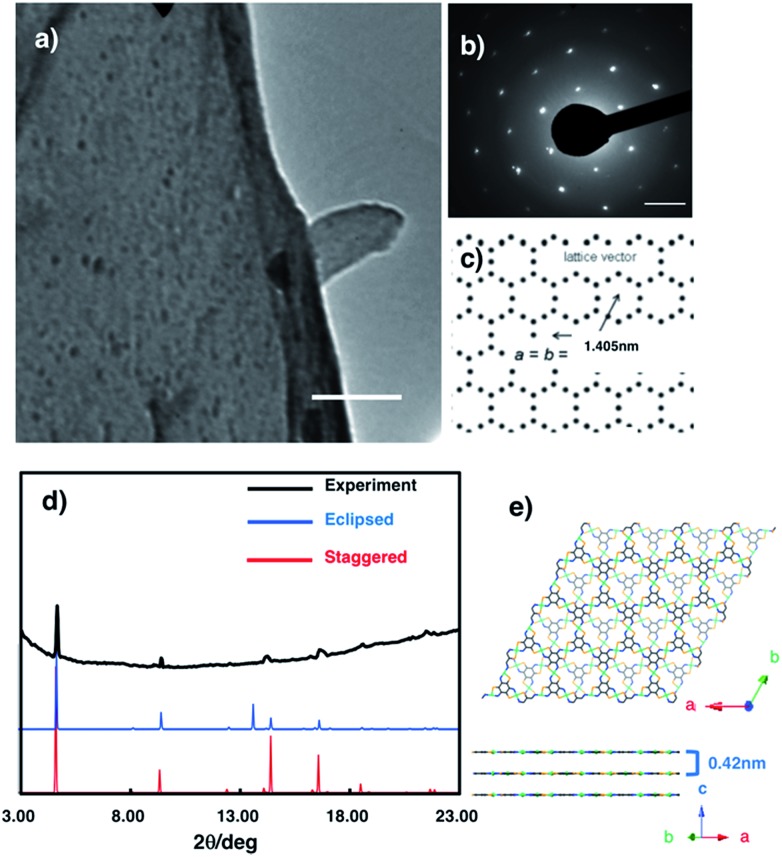
(a) HR-TEM images of **NiAT**. Scale bars represent 500 nm. (b) SAED pattern of **NiAT**. (c) Hexagonal two-dimensional lattice that gives the SAED pattern. Scale bar represents 2 nm^–1^. (d) Experimental and simulated GIXD patterns of **NiAT**. (e) Model structures of the staggered stacking structure.

The crystal structure of multi-layered **NiAT** was analysed using diffraction techniques. High-resolution transmission electron microscopy (HR-TEM) showed its sheet-like morphology (Fig. 4a), and selected-area electron diffraction (SAED) found a hexagonal diffraction pattern (Fig. 4b and S6†), implying a hexagonal lattice with a cell length of 1.41 nm. Grazing incidence X-ray diffraction (GIXD) analysis using synchrotron radiation (*λ* = 1.00 Å) found prominent peaks (Fig. 4d) for **NiAT** that matched the diffraction pattern of the staggered stacking structure (*P*3, *a* = *b* = 1.405 nm and *c* = 0.84 nm) rather than that of the eclipsed stacking structure (absence of an in-plane intense peak at 2*θ* = 13.7°) (Fig. 4e and S7†). Additionally, multiple interlayer distance patterns were subjected to both staggered and eclipsed stacking structures to identify that the interlayer distance of 0.42 nm of staggered stacking structure agrees with the experimental observation (Fig. S7a,† examples of simulation using different interlayer distance). Moreover, slipped-parallel (AB) orientation pattern simulations were performed by fixing an interlayer separation of 0.42 nm with different stacking angels, which again supported the staggered stacking (Fig. S7b,† examples of simulation using different stacking angels). By comparing the observed GIXD profile with the simulated results, the sharp peaks at 2*θ* = 4.7°, 9.4°, 12.5°, and 21.7° were identified as the in-plane 1 0 0, 2 0 0, 3 1 0, and 5 1 0 diffractions, respectively. GIXD indicated that the present nanosheet had a crystalline hexagonal lattice with an in-plane distance of 1.41 nm and interlayer distance of 0.42 nm.

The **NiAT-M** and **NiIT-M** complexes are reversibly interconverted by a 2H^+^–2e^–^ reaction;^66^ therefore, interconversion of **NiAT** and **NiIT** was examined. The conversion of **NiAT** to **NiIT** was performed by treating **NiAT** suspended in water with a base (NEt_3_) followed by adding ferrocenium tetrafluoroborate as an oxidizing agent. The conversion was reversed by the addition of acetic acid followed by the reducing agent, decamethylcobaltocene. This was confirmed directly by monitoring the characteristic N–H stretching signals in the IR spectra (Fig. S8†). The double N–H stretching peaks changed to a single peak upon the chemical oxidation of **NiAT**, indicating the formation of **NiIT**, and the double peaks reappeared upon the chemical reduction of **NiIT**, forming **NiAT**. Overall, these results demonstrate that **NiAT** and **NiIT** are chemically interconvertible without decomposition or a change in shape.

**Fig. 5:**
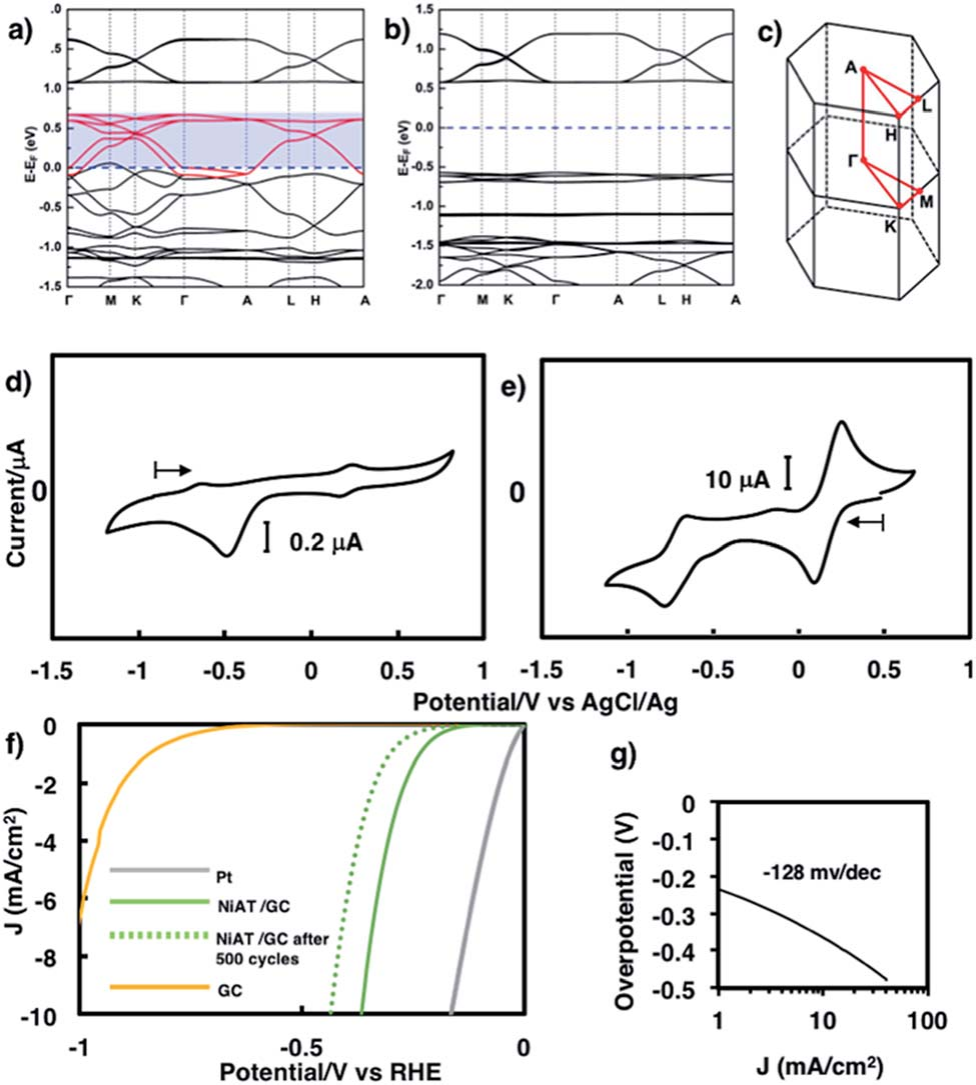
First-principles band structures of (a) **NiAT** and (b) **NiAT**. (c) First Brillouin zone and high-symmetry *k*-points of **NiIT** and **NiAT**. (d) Cyclic voltammogram of (d) **NiAT** and (e) **NiIT** in 0.1 M KCl aq. at a scan rate of 50 mV s^–1^. (f) Linear sweep voltammograms for HER reaction with Pt, **NiAT**/GC, and GC in 0.05 M H_2_SO_4_ and (g) its Tafel slope.

We previously found that pelletized **NiIT** showed a semiconducting nature with the activation energy of 41 meV and the electrical conductivity of 1 × 10^–1^ S cm^–1^ at 298 K.^65^ Here, we measured the conductivity of pelletized **NiAT**, finding it to be close to an insulator with the activation energy of 113 meV and the conductivity of 3 × 10^–6^ S cm^–1^ at room temperature (Fig. S9†). This implies that **NiAT** functions as a redox switch of conductivity. The lower conductivity of **NiAT** was because there were no unpaired free electrons delocalized laterally in the sheet, in contrast to **NiIT**. The calculated band structures of **NiIT** (Fig. 5a) and **NiAT** (Fig. 5b) clearly show a typical kagome band, characterized by a set of Dirac bands and a flat band mostly derived from Ni-d_*x*^2^–*y*^2^_ orbitals. The band structure of **NiIT** indicates a metallic state with high electron density of states at the Fermi level, whereas that of **NiAT** shows an insulating state with the Fermi level lying inside a gap larger than 1.0 eV. The effect of adding one H atom per N atom to the **NiAT** sheet is to dope the system with electrons. There are six bands in **NiIT** between the Fermi level and the band gap above (shaded region in Fig. 5a), which are mainly ascribed to the p_*z*_ orbitals of C, N, and S. **NiAT** has 12 more electrons (one contributed by every H atom) than **NiIT** per unit cell, leading to all the six empty bands being filled, shifting the Fermi level into the band gap. In addition to doping electrons, after adding H, the N valence orbitals are fully filled with a closed shell, so that electrons can hardly hop through the N sites. This effectively decreases the long-range hopping interaction between carbon rings, which in turn narrows the bandwidth and increases electron localization. Moreover, molecular orbitals by DFT calculation showed the frontier orbitals of **NiIT** to be greatly delocalized over the entire ring, whereas those of **NiAT** are strongly localized (Fig. S11†). This also indicates much larger charge delocalization in **NiIT** than **NiAT**, consistent with the band structure calculation results. These theoretical results suggest that **NiIT** has a much higher electrical conductivity than **NiAT**, supporting the experimental observation.

Cyclic voltammograms of **NiAT** and **NiIT** in 0.1 M KCl are shown in Fig. 5d and e, respectively. **NiIT** had two quasi-reversible pairs at 0.24 and –0.66 V (Fig. 5e), suggesting that it underwent two successive, reversible one-electron reductions and two successive, one-electron oxidations. **NiAT** exhibited an irreversible peak at –0.48 V, probably caused by hydrogen migration from the amino group to the nickel centre to form a Ni–H bond.^66^ The utilization of nanostructures as electrocatalysts for energy conversion technologies are intensively studied because their structures allowed for the sufficient exposure of well-defined active sites.^55^,^58^,^63^,^64^,^67^,^68^ The electrocatalytic activity of **NiAT** for the HER was examined because the **NiAT-M** complex exhibits higher catalytic ability for the HER than bis(diamino)nickel or bis(dithiolato)nickel.^66^**NiAT** on glassy carbon (GC) protected with Nafion® was used as an electrocatalyst for the HER in acidic and neutral aqueous solutions. As the pH of the solution decreased, the catalytic efficiency increased (Fig. S12†). At pH = 1.3, the onset potential for hydrogen evolution was –0.15 V *vs.* a reversible hydrogen electrode (RHE) with an operating potential of –0.37 V at 10 mA cm^–2^ (Fig. 5f) and a Tafel slope of 128 mV dec^–1^ (Fig. 5g). The HER exchange current density (*j*_0_) for the **NiAT** catalysts obtained from the Tafel plots using the extrapolation method was 0.04 mA cm^–2^ (Fig. S13†). The Tafel slope in the plots is 128 mV dec^–1^, which was further confirmed by electrochemical impedance spectroscopy (EIS, Fig. S14†). The charge-transfer Tafel slope derived from the linear fit of the plot of log *R*_ct_*versus* overpotential was 131 mV dec^–1^ close to the value obtained by the Tafel plots, indicating not only a charge transfer rate defining step but also a swift electron transfer characteristic of the **NiAT** catalyst in the HER.^69^ The turnover frequency (TOF), indicating the number of H_2_ molecules generated per second per active site, was calculated to be 0.1 s^–1^ at overpotential of 300 mV, using electrochemical surface area (ECSA, Fig. S15†) of the **NiAT** sheet by testing the electrochemical double layer capacitance (*C*_dl_) (Fig. S16†). Compared with similar materials such as Co-benzenehexathiol and Co-triphenylenehexathiol complex nanosheets for HER catalysts,^64^ bis(aminothiolato)nickel motif outperformed bis(dithiolato)cobalt motif in many aspects. Moreover, **NiAT** exhibited relatively stable performance during 500 HER cycles (Fig. S17,† potential cycling in the range of 0.27 to –0.93 V *vs.* RHE), XPS of **NiAT** showed that the component ratios and valence state remained unchanged after 500 HER cycles demonstrating the durability in acidic conditions (Fig. S18†). These results show that **NiAT** is an efficient, durable electrocatalytic cathode material for hydrogen generation from water.

## Conclusions

In conclusion, we synthesized a crystalline CONASH containing **NiAT** moieties using the liquid–liquid and gas–liquid interfacial reactions. The morphology, composition, and crystal structure of **NiAT** were characterized by AFM, FE-SEM, XPS, ATR-IR, HR-TEM, and GIXD. **NiAT** is an insulator and interconvertible to the conducting **NiIT** nanosheet by a proton-coupled redox reaction. These drastic changes in electrical conductivity were explained by the theoretical calculation of band structures. **NiAT** showed remarkable performance as an electrochemical HER catalyst. These results demonstrate a rare example of a CONASH acting as a redox switch for conductivity and as an electrocatalyst. Utilization for FET and other catalytic activities and energy storage abilities of this dual CONASH are currently being investigated.

## Methods

### Materials

All starting material and solvents were purchased from Tokyo Chemical Industry Co., Ltd. Dichloromethane was purified with a glass contour solvent dispensing system (Nikko Hansen & Co., Ltd.). Water was purified using the Milli-Q purification system (Merck). 1,3,5-Triaminobenzene-2,4,6-trithiol **(L)**, mononuclear complex bis(1-aminobenzene-2-thiolato)nickel(ii) [**NiAT-M**], mononuclear complex bis(1-iminobenzene-2-thiolato)nickel(ii) [**NiIT-M**] and were prepared according to the literature.^66^,^70^

### Substrate preparation

HOPG was purchased from Alliance Biosystems, Inc. (Grade SPI-1 10 × 10 × 2 mm) and cleaved with adhesive tape prior to use. Silicon wafers (p-doped with a carrier concentration of 3 × 10^18^ cm^–3^ and resistivity of 1–10 ohm cm) with thermally-grown 100 nm-thick SiO_2_ were purchased from Yamanaka Semiconductor and cut into squares (10 × 10 mm). 1,1,1,3,3,3-Hexamethyldisilanaze (HMDS)-modified SiO_2_/Si substrates were prepared by depositing HMDS on SiO_2_/Si substrates, keeping them under vacuum for 10 min, and then placing them in a flow of Ar. The HMDS-modified SiO_2_/Si substrates were kept in anhydrous ethanol.

### Preparation of multi-layer **NiAT**

Under an Ar atmosphere, a degassed aqueous solution (10 mL) containing 2.4 × 10^–5^ mol L^–1^ of **L**, and organic layer of a dichloromethane solution of 0.5 mM Ni(acac)_2_ (10 mL). The organic layer was overlaid slowly with the aqueous **L** solution. After standing in an Ar atmosphere for 10 days, a yellow nanosheet was obtained at the aqueous/organic interface. After the removal of the aqueous and organic phases, multi-layer **NiAT** was washed thoroughly with water, ethanol, and dichloromethane, and dried *in vacuo* at 120 °C. **NiAT** could be oxidized under long time explosion into air, thus all characterizations and measurements took suitable oxygen segregation treatments. Elemental analysis for NiC_4_H_4_N_2_S_2_: calcd: C: 23.66%; H: 2.02%; N: 13.81%. Found: C: 23.36%; H: 2.32%; N: 14.12%.

### Preparation of single-layer **NiAT**

A dilute dichloromethane (10 μL)-ethyl acetate (1 μL) solution of Ni(acac)_2_ (0.5 mM) was gently dropped onto an aqueous solution of **L** (35 μM) at ambient temperature using a micro syringe. After spontaneous evaporation of the organic solvent, the reaction system was left undisturbed, so that single-layer **NiAT** was produced at the air–liquid interface. The nanosheet was then transferred onto substrates using the Langmuir–Schaefer method.

### Characterization

Optical microscope images were taken using VHX-100 (Keyence Corporation). FE-SEM images were collected using a scanning electron microscope (JSM-7400 FNT, JEOL). The samples were prepared by depositing an ethanol suspension of **NiAT** on HMDS-modified SiO_2_/Si substrates. TEM images were recorded with a transmission electron microscope (HF-2000, Hitachi) equipped with a AMT-CCD camera (HISCO, AMT552) at 75 kV. The TEM samples were prepared by depositing an ethanol suspension of **NiAT** on a carbon film supported by a copper grid. GIXD data were obtained using synchrotron radiation (*λ* = 1.00 Å) at BL45XU in SPring-8, Japan. Diffractions from a sample were detected using a Pilatus3X 2M detector. Recorded diffraction images were integrated along the Debye–Scherrer ring using a FIT2D software, affording a one-dimensional intensity profile. To prepare the samples, **NiAT** was deposited on HMDS-modified SiO_2_/Si substrates. ATR-IR spectra were recorded using an IR spectrometer (FT/IR-6100, JASCO) at room temperature under vacuum. The samples were prepared by depositing **NiAT** on HMDS-modified SiO_2_/Si substrates. XPS data were obtained using an XPS microprobe (PHI 5000 VersaProbe, ULVAC-PHI, Inc.). Al Kα (15 kV, 25 W) was used as the X-ray source, and the beam was focused on a 100 μm^2^ area. The spectra were analyzed with MultiPak (MultiPak Version 9.2.0.5 ULVAC-PHI, Inc.), and standardized using the C (1s) peak at 284.8 eV. Atomic force microscopy measurements were carried out using a scanning probe microscope (5500, Agilent Technologies) under ambient conditions high-amplitude mode (tapping mode) with a silicon cantilever probe (PPP-NCL, Nano World).

### SAED and GIXD simulation

To reproduce the obtained SAED and 2D WAXS pattern (Fig. 3d), two types of 3D lattices comprising piles of single-layer **NiAT** were considered. The two lattices include eclipsed (AA) and staggered (AA^–1^) stack models, because 6-fold symmetry is required to reproduce the hexagonal diffraction pattern in SAED. First, the atomic arrangement of single-layer **NiAT** was optimized using density-functional theory implemented in performed on Gaussian 09:^71^ all the optimized structures were obtained at the B3LYP level of theory. As the basis sets, 6-31G* is used. The optimized cell was then subjected so as to reproduce the observed diffraction data. The eclipsed and staggered stack models were then constructed by using space group of *P*3 and *P*3, respectively. Slipped-parallel (AB) orientations pattern simulations were performed using eclipsed (AA) stack models fixing an interlayer separation of 0.42 nm with different stacking angles *β*. The SAED and GIXD patterns were simulated by implementing CrystalMaker 2.6.3, SingleCrystal 2.3, and CrystalDiffract 6.5.5 (CrystalMaker Software Ltd).

### Electrical properties

Electrical conductivity data were collected using the van der Pauw method.^72^**NiAT** (2.1 mg) prepared using conditions of described in Preparation of multi-layer **NiAT** were grinded then pressed with a pressure of 500 kg cm^–2^ to form self-standing films. The pelletized films were placed on fluoropolymer boards and attached to gold wires using carbon paste (Fujikura Kasei Co., Ltd.). The O_2_ prevention procedures are applied to every subjectable step.

### Band structure calculation

The first-principles band structure calculations based on density-functional-theory method were performed in the framework of the projector augmented wave approach and the generalized gradient approximation of Perdew–Burke–Ernzerhof for the exchange–correlation potentials, as implemented in Vienna *ab initio* simulation package.^73^,^74^ Self-consistent calculations were carried out with an energy cut off of 400 eV on a 5 × 5 × 7 *Γ*-centered *K*-point mesh. All atoms were fully relaxed in the structural optimizations until the atomic forces were smaller than 0.01 eV Å^–1^. Electron spin polarization was also considered. The experimental lattice constants of **NiAT** and **NiIT** with AB-stacking pattern were adopted for the band structure calculations.

### Electrochemical methods

Electrochemistry experiments were carried out using an electrochemical analyzer (650DT, ALS). A platinum wire served as the auxiliary electrode, and the reference electrode was an Ag/AgCl electrode (silver wire immersed in 0.1 M Bu_4_NClO_4_/0.01 M AgClO_4_/CH_3_CN), calibrated with a ferrocenium/ferrocene redox potential. To make the working electrode, **NiAT** (1.7 mg) was placed on 10 mm φ aluminum foil to cover entire surface, another 10 mm φ carbon paper was put on top to make a “sandwich”, then pressed with a pressure of 500 kg cm^–2^. For the HER, a 3 mm φ GC working electrode was used. Before use, the GC electrodes were polished using aqueous alumina suspensions on felt polishing pads. The potentials for the HER in this study refer to those of the reversible hydrogen electrode (RHE) obtained by adding E(SCE) + 0.059 pH. The aqueous solutions used in the electrochemical experiments were prepared as follows. For cyclic voltammograms of **NIAT**, KCl (0.1493 g) was dissolved in water (20 mL) to make 0.1 M KCl aqueous solution. For the pH 1.3 solution, H_2_SO_4_ (0.0534 mL, *c* = 98%) was dissolved in water (20 mL). The solutions were deoxygenated with pure Ar before measurement. The catalyst ink was prepared by suspending **NiAT** (0.2 mg) in ethanol (4 mL). The catalyst ink was pipetted onto the GC surface to achieve the desired catalyst loading. Then, 7 μL of 5% Nafion® solution in ethanol was drop-cast on top to protect the **NiAT** film. The HER was measured by cyclic voltammetry with a sweep rate of 50 mV s^–1^. For comparison, the same GC electrode was re-polished after drop casting the catalyst, redrop the same amount of Nafion without catalyst and measured in the same aqueous solution to give a naked GC HER result. Moreover, a 2 mm φ Pt electrode was also measured for comparison.

## Conflicts of interest

There are no conflicts of interest to declare.

## Supplementary Material

Supplementary information
