# Supplementary material for: Bis(aminothiolato)nickel nanosheet as a redox switch for conductivity and an electrocatalyst for the hydrogen evolution reaction
Source: Chem Sci. 2017 Oct 3;8(12):8078–85. doi: 10.1039/c7sc02688a (PMC5855133; doi:10.1039/c7sc02688a)
Supplement: Supplementary file 1 [file SC-008-C7SC02688A-s001.pdf]

Electronic Supplementary Material (ESI) for Chemical Science.
This journal is © The Royal Society of Chemistry 2017

Supporting Information

Bis(aminothiolato)nickel Nanosheet as a Redox Switch for Conductivity and an Electrocatalyst for the Hydrogen Evolution Reaction

Xinsen Sun,[a] Kuo-Hui Wu,[a] Ryota Sakamoto,[a] Tetsuro Kusamoto,[a] Hiroaki Maeda,[a] Xiaojuan Ni,[b] Wei Jiang,[b] Feng Liu,[b] Sono Sasaki,[c,d] Hiroyasu Masunaga[e] and Hiroshi Nishihara[a*]

[a]Department of Chemistry, School of Science, The University of Tokyo, 7-3-1, Hongo, Bunkyo-ku, Tokyo, 113-0033 Japan

[b]Department of Materials Science and Engineering, University of Utah, Salt Lake City, UT 84112, USA

[c] Faculty of Fiber Science and Engineering, Kyoto Institute of Technology, Matsugasaki Hashikami-cho 1, Sakyo-ku, Kyoto 606-8585, Japan

[d]RIKEN SPring-8 Center, Kouto 1-1-1, Sayo-cho, Sayo-gun, Hyogo 679-5148, Japan

[e]Japan Synchrotron Radiation Research Institute (JASRI)/SPring-8, 1-1-1 Kouto, Sayo-cho, Sayo-gun, Hyogo 679-5198, Japan

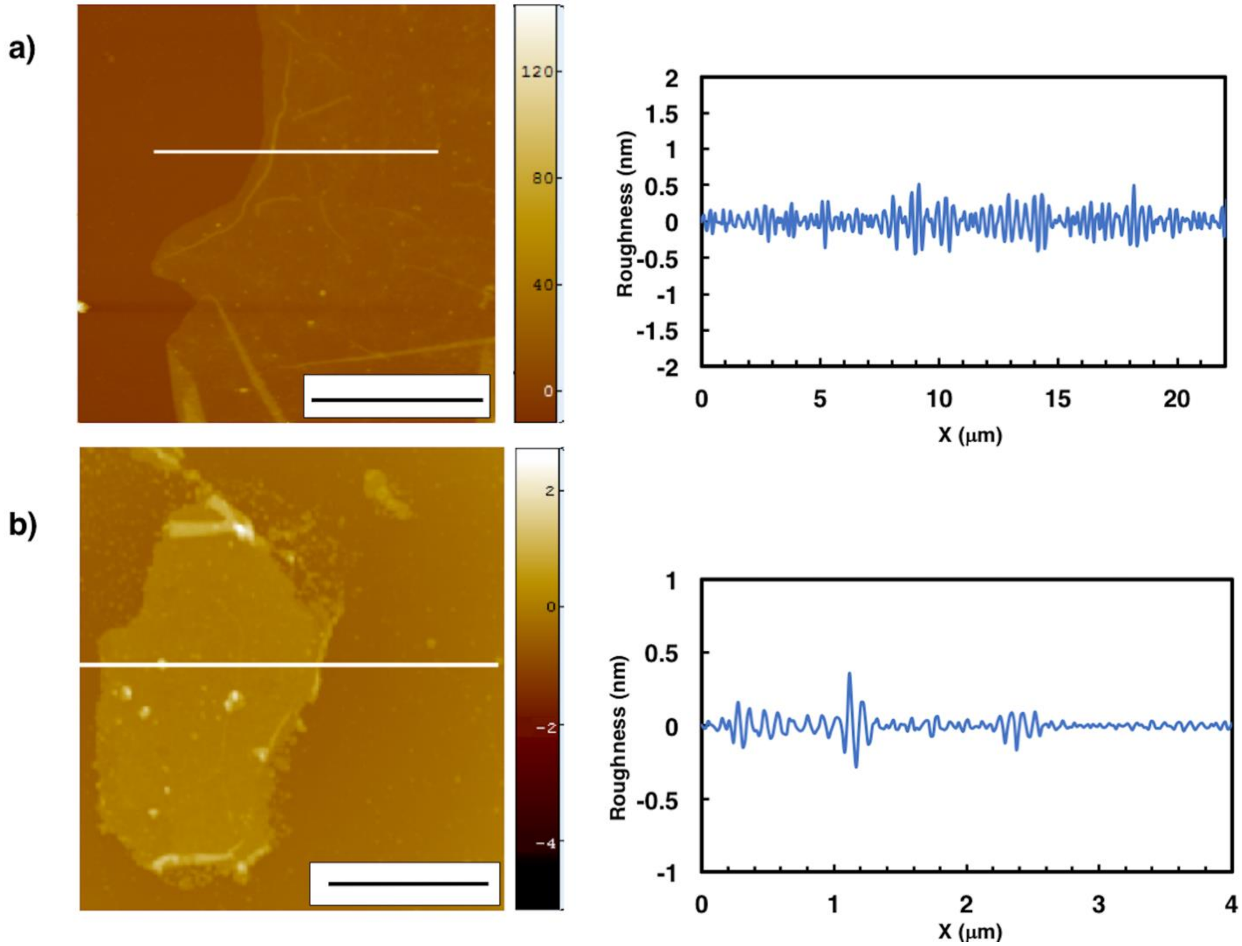

Fig. S1. **a)** AFM image on HMDS/Si(111) with height distribution indication and its roughness analysis along the cross-section white line of the multi-layer**.** Scale bars represent 10 μm. **b)** AFM image on HMDS/Si(111) with height distribution indication and its roughness analysis along the cross-section white line of the single-layer**.** Scale bars represent 2 μm.

IR spectra of **NiIT, NiAT** and ligand **L** (Fig. S2)

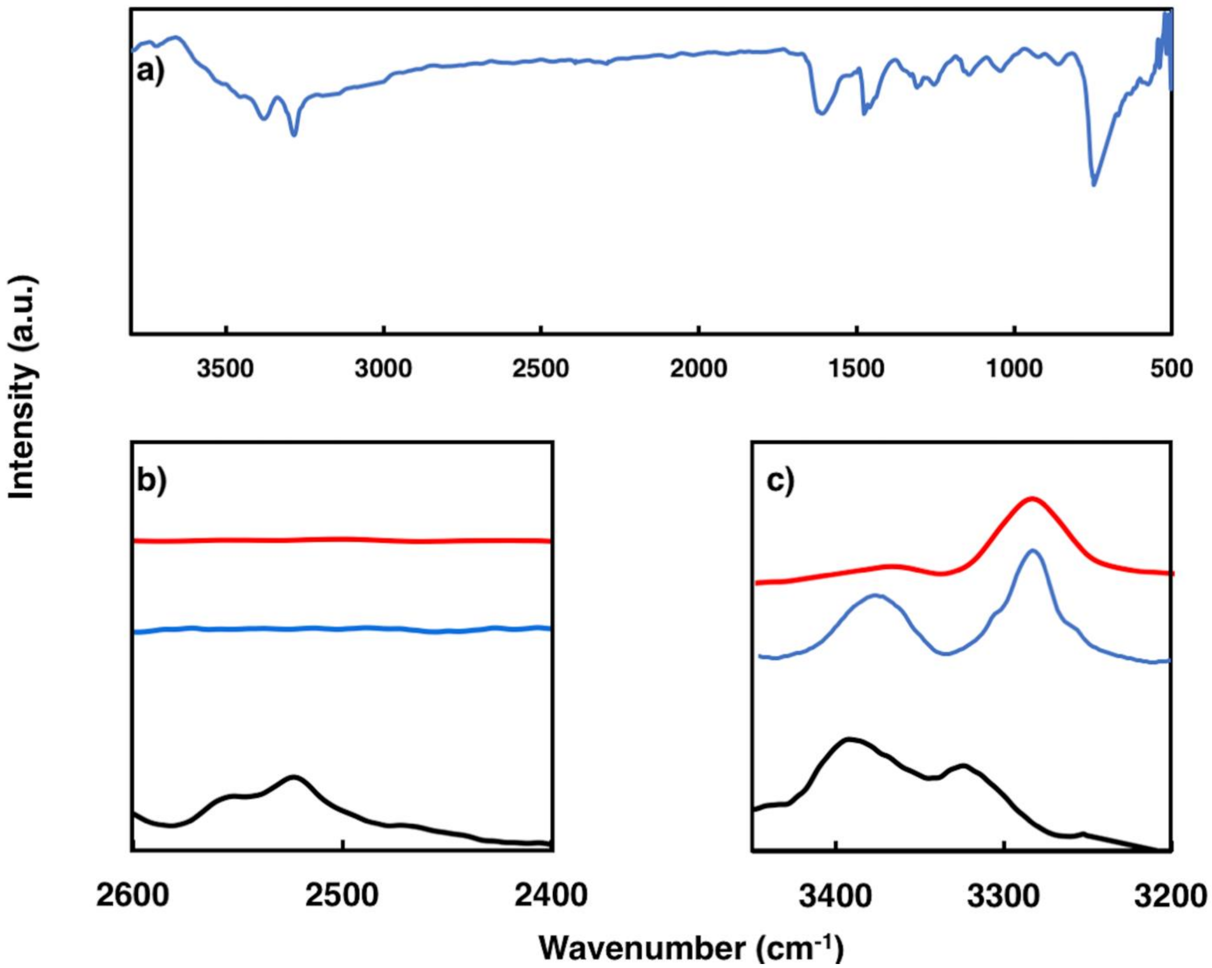

Fig. S2. **a)** Full IR spectra using ATR method of **NiAT**. Comprising of **NiIT** (red), **NiAT** (blue) and **L** (black). **b)** Focusing S-H stretching. **c)** Focusing on N-H stretching.

XPS of ligand **L** (Fig. S3)

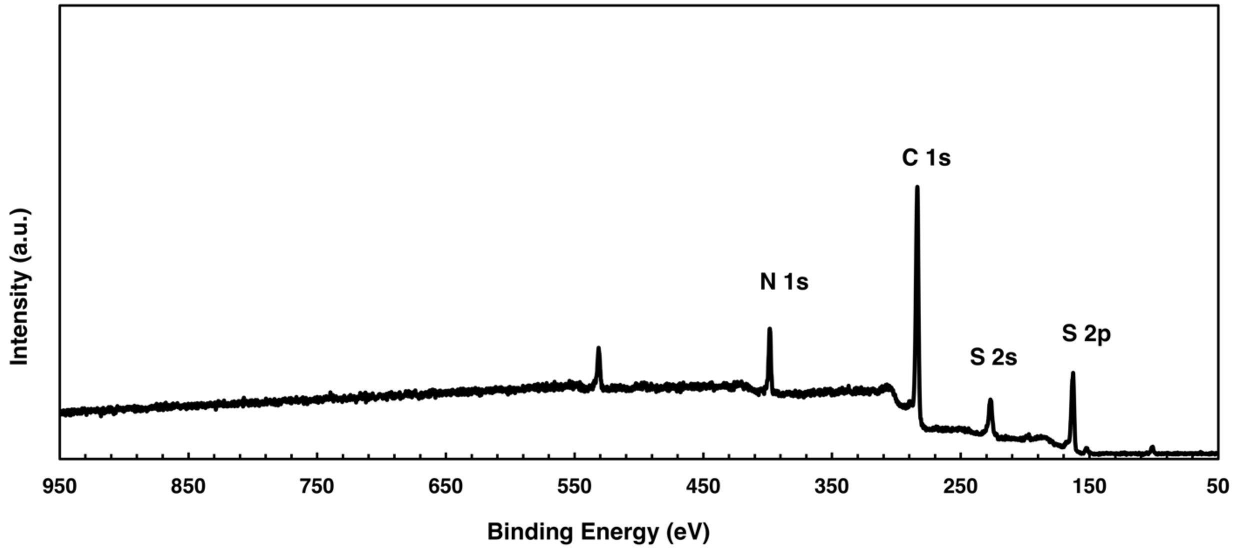

Fig. S3. Wide-scan XPS of ligand **L.**

XPS of mononuclear **NiAT-M** (Fig. S4)

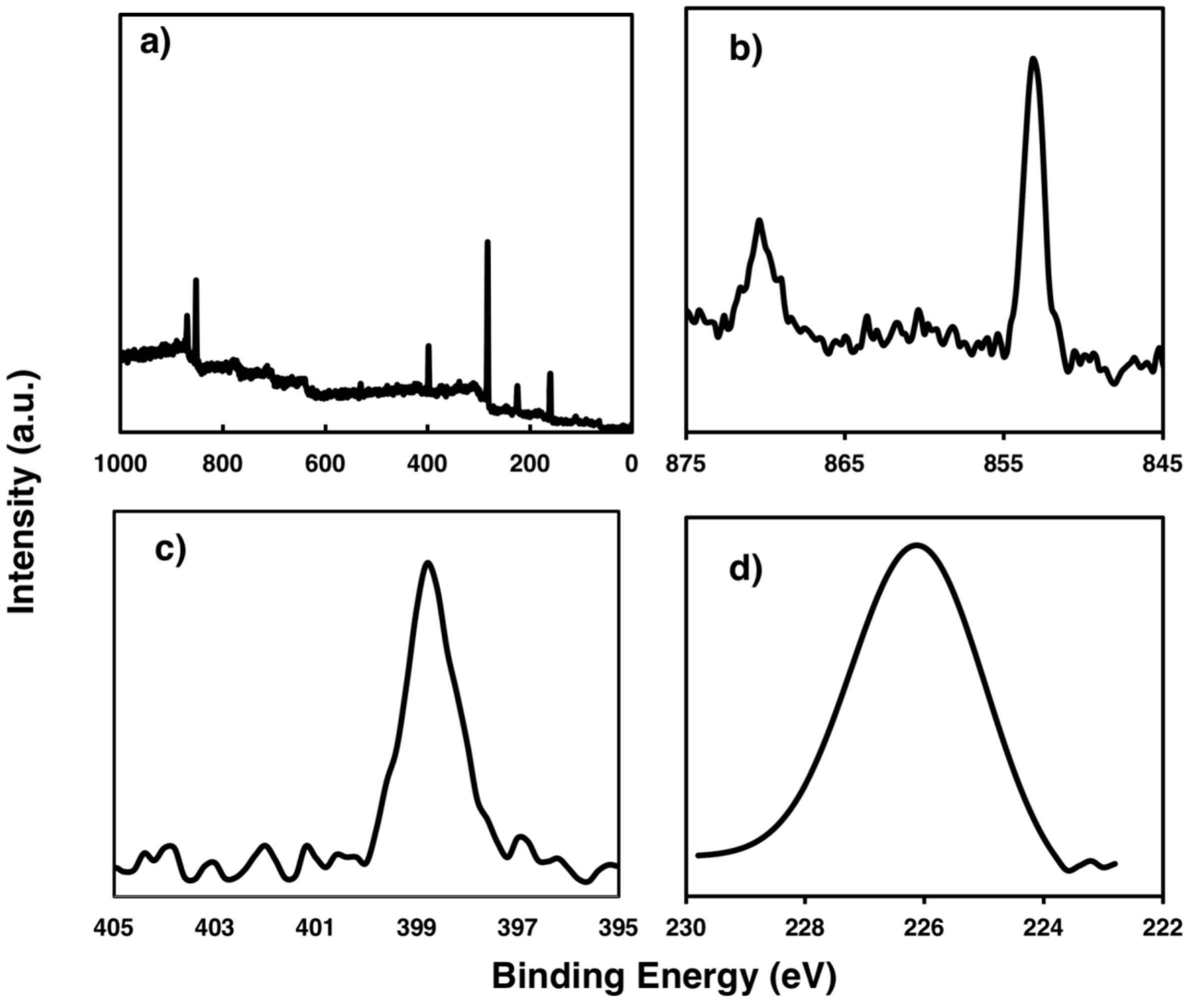

Fig. S4. **a)** Wide-scan XPS of **NiAT-M**. Narrow-scan XPS focusing on **b**) the Ni 2p region, **c**) the N 1s region, **d**) the S 2s region

XPS of mononuclear **NiIT-M** (Fig. S5) and **NiIT** (Fig. S6)

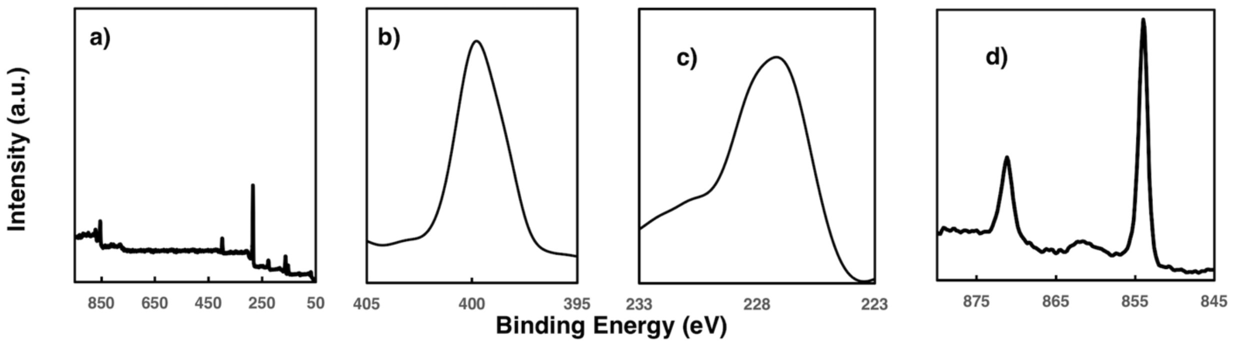

Figure S4. **a)** Wide-scan XPS of **NiIT-M**. Narrow-scan XPS focusing on **b**) the N 1s region, **c**) the S 2s region. **d**) the Ni 2p region,

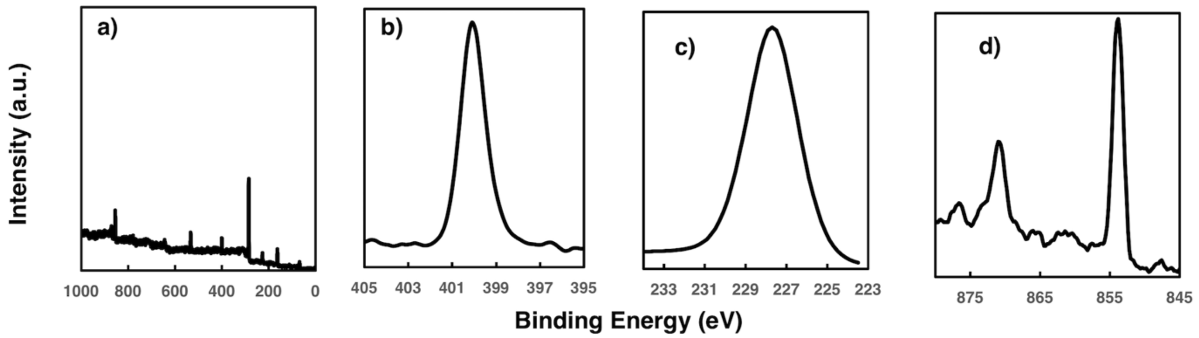

Fig. S5. **a)** Wide-scan XPS of **NiIT**. Narrow-scan XPS focusing on **b**) the N 1s region, **c**) the S 2s region. **d**) the Ni 2p region.

Table S1. XPS of N 1s and S 2s peak regions combined table

	**N 1s/eV**	**S 2s/eV**
L	400.2	227.8
NiIT-M	400	227.5
NiAT-M	399.1	226.2
NiIT	399.7	227.4
NiAT	398.9	226.5

SAED pattern of **NiAT** (Fig. S6)

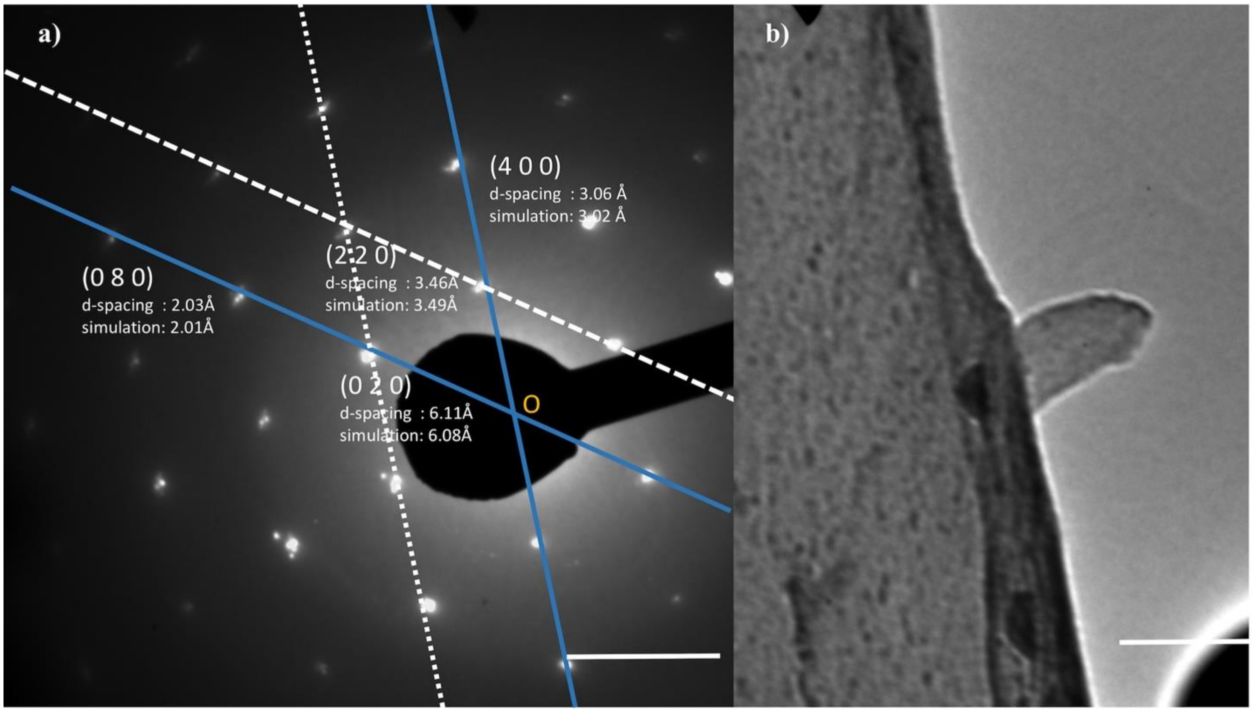

Fig. S6. **a)** SAED pattern of **NiAT**, scale bar 2nm^{-1} **b)** TEM image which showed the SAED pattern, scale bar: 500 nm.

Simulation structure of NiAT (Fig. S7)

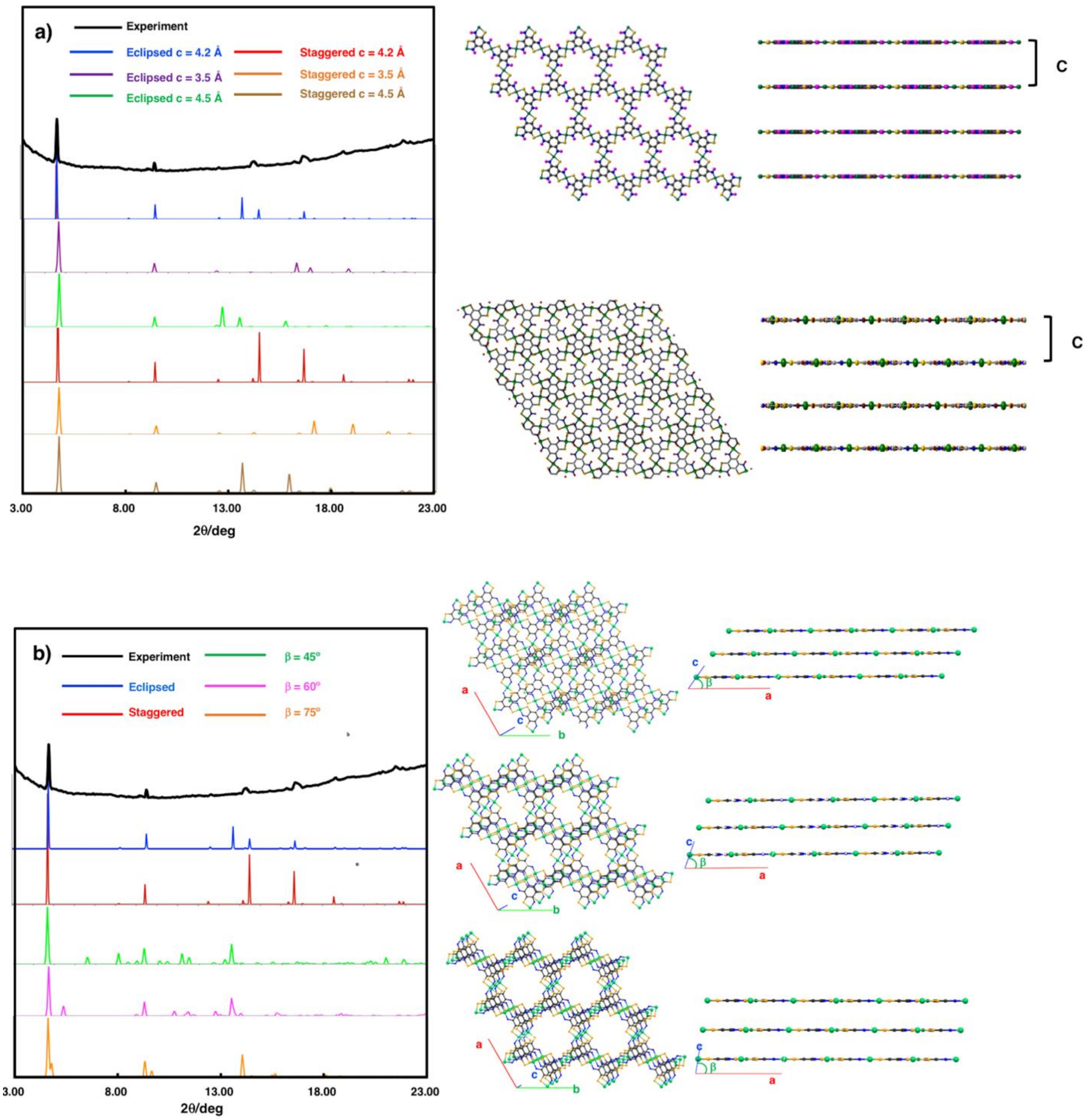

Fig. S7. **a)** The simulation structure using for eclipsed pattern. Space group: *P*3. Unit cell Length: *a* = *b* = 1.41 nm. The interlayer distance, c, using 0.42 nm, 0.35 nm and 0.45 nm. And the simulation structure using for staggered pattern. Space group: *P*–3, Unit cell Length: *a* = *b* = 1.405 nm. The interlayer distance, c, using 0.42 nm, 0.35 nm and 0.45 nm. **b)** The simulation structure using for eclipsed pattern. Space group: *P*3. Unit cell Length: *a* = *b* = 1.41 nm. The interlayer distance is 0.42 nm. Modifying β angel to changing the stacking pattern.

Chemical conversion between **NiIT** and **NiAT** (Fig. S8)

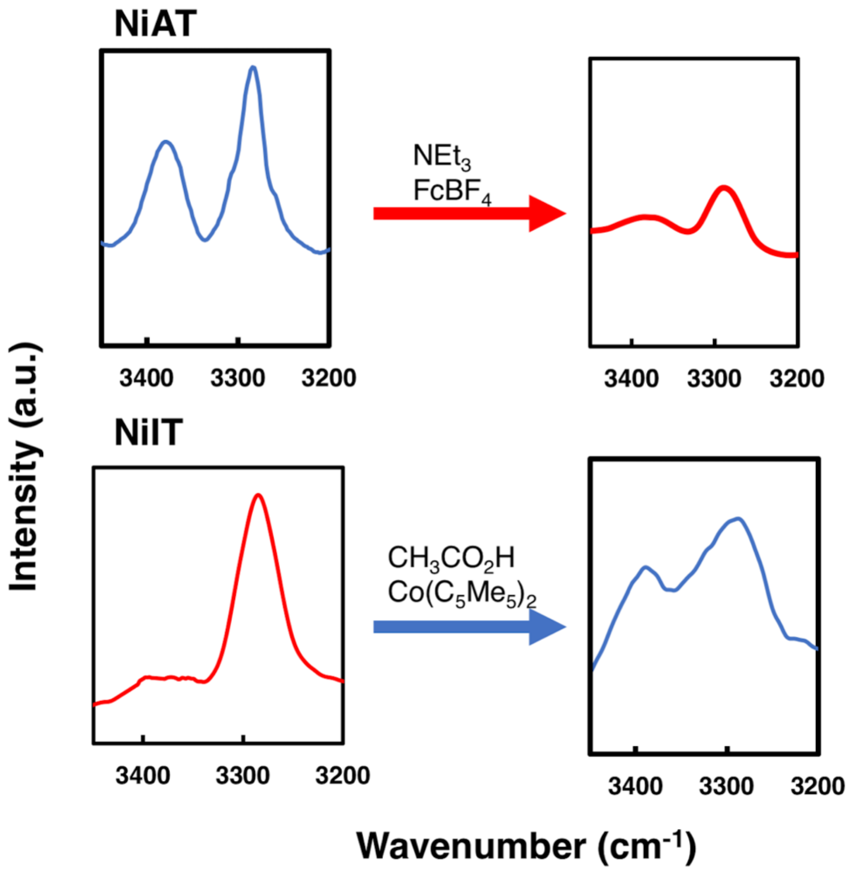

Fig. S8. **a** IR of **NiAT** before and after chemical oxidization. **b** IR of **NiIT** before and after chemical reduction.

Temperature dependent conductivity measurement of **NiAT** (Fig. S9)

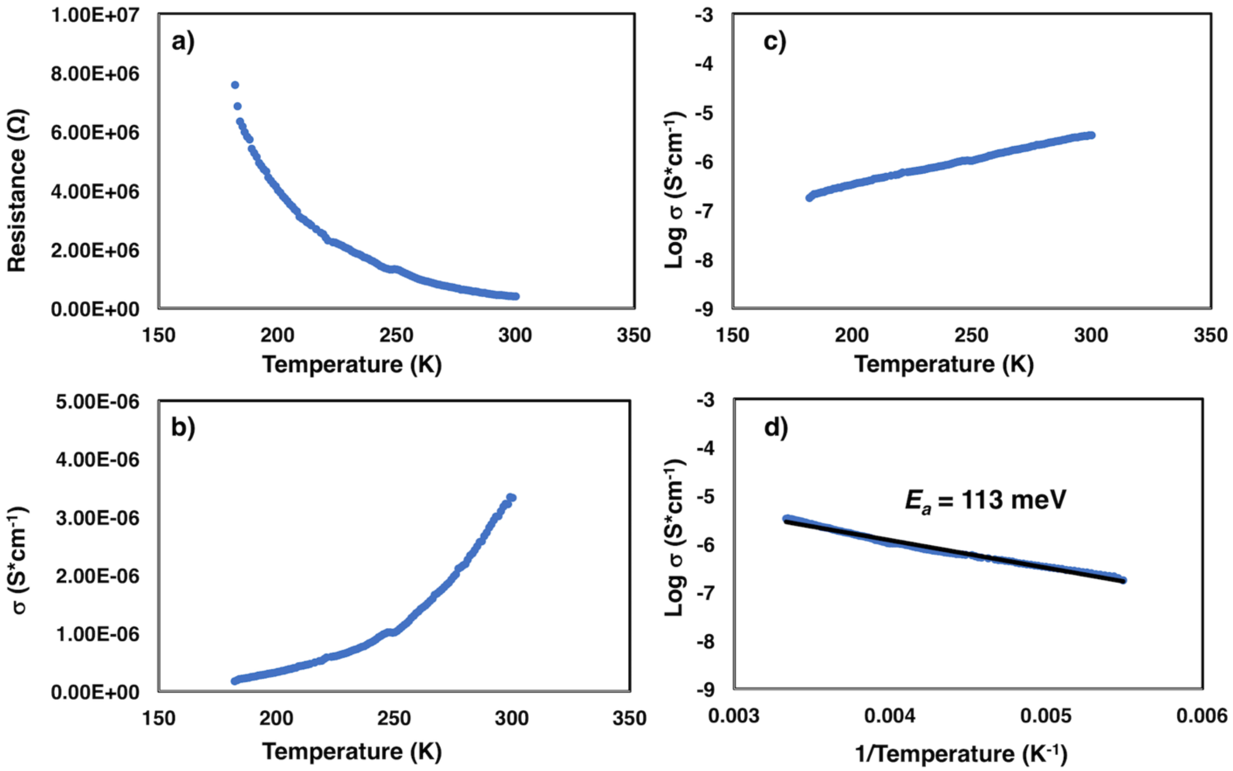

Fig. S9. **a**, Temperature depended resistance plot of **NiAT. b, c**, **d**, Temperature depended conductivity plot of **NiAT.**

Structures of **NiIT** and **NiAT** were used to calculate bands (Fig. S10)

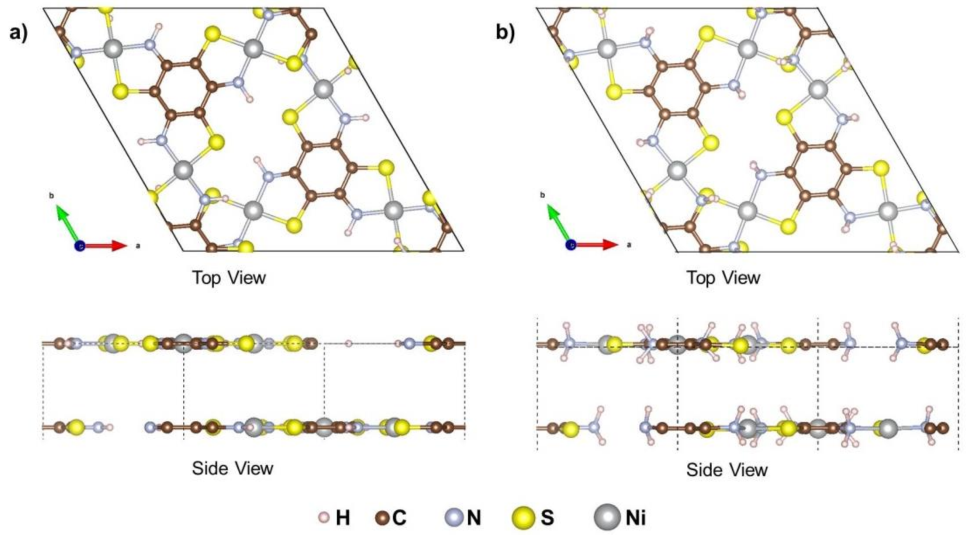

Fig. S10. Schematic representations of (a) **NiIT** and (b) **NiAT** structures to calculate bands.

MOs of **NiIT** and **NiAT** (Fig. S11)

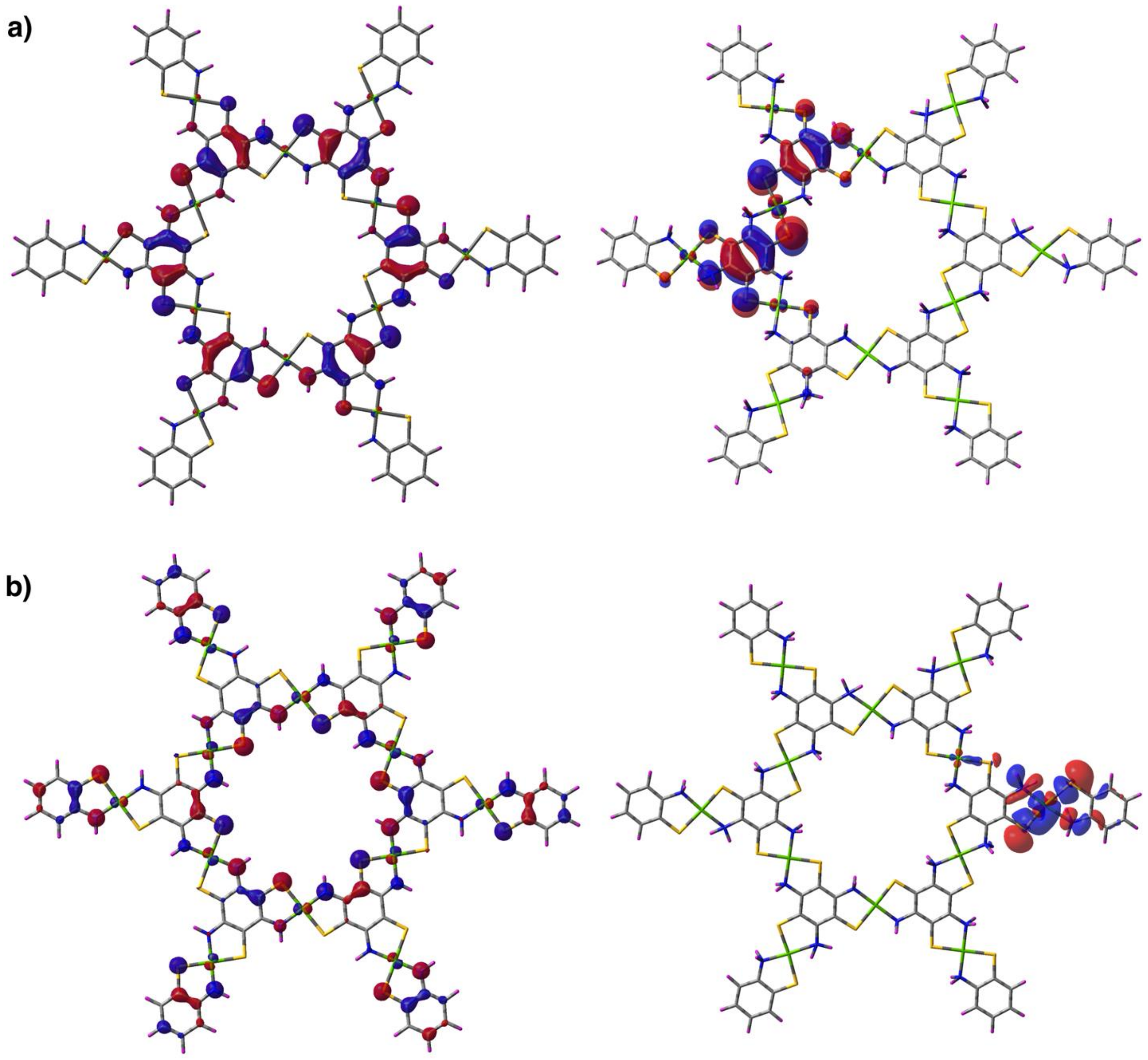

Fig. S11. **a)** Calculated MOs of **NiIT** (left) and **NiAT** (right) at HOMO level. **b)** Calculated MOs of **NiIT** (right) and **NiAT** (left) at LUMO level.

HER of **NiAT** at different pH value (Fig. S12)

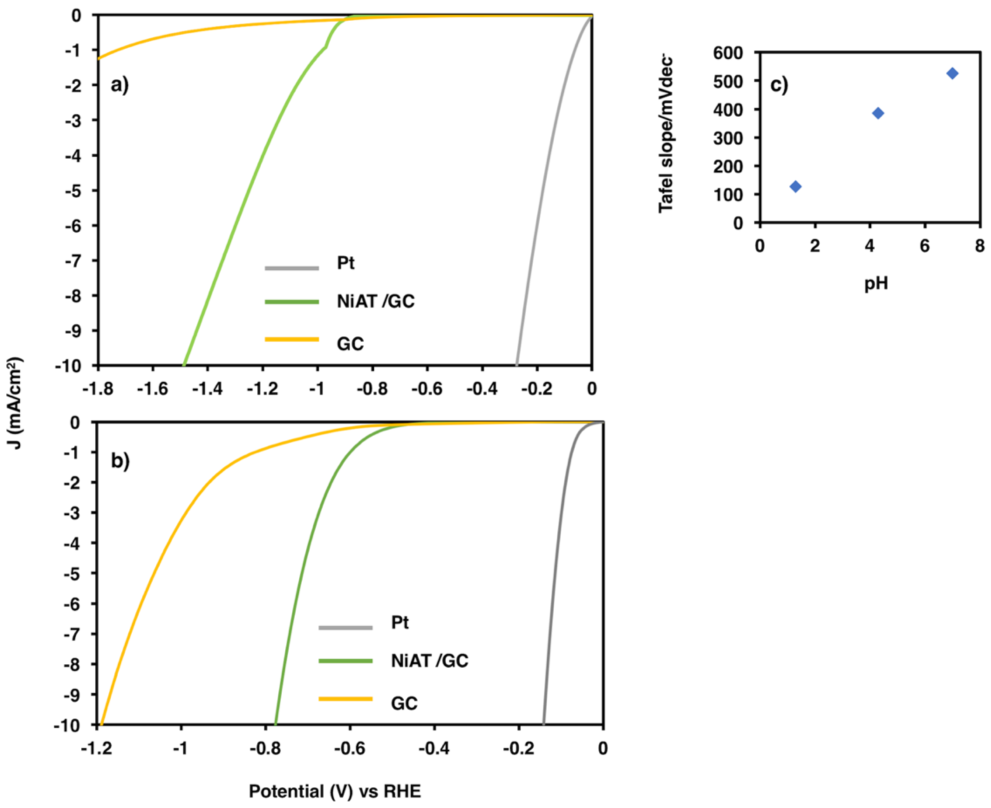

Fig. S12. **a**) Linear sweep voltammograms for HER reaction performed by Pt, **NiAT**/GC, GC under pH7 condition. **b**) Linear sweep voltammograms for HER reaction performed by Pt, **NiAT**/GC, GC under pH3.1 condition. **c)** And tafel plot.

Tafel slop and exchange current density (j_0) of **NiAT** (Fig. S13)

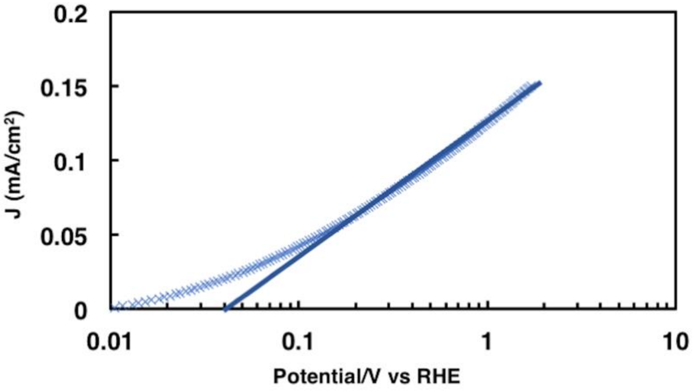

Fig. S13. Tafel slop and exchange current density (j_0) of **NiAT**, j_0 is estimated by linear fitting of the polarization curves using the Butler-Volmer equation.

Tafel slop and exchange current density (j_0) of **NiAT** (Fig. S14)

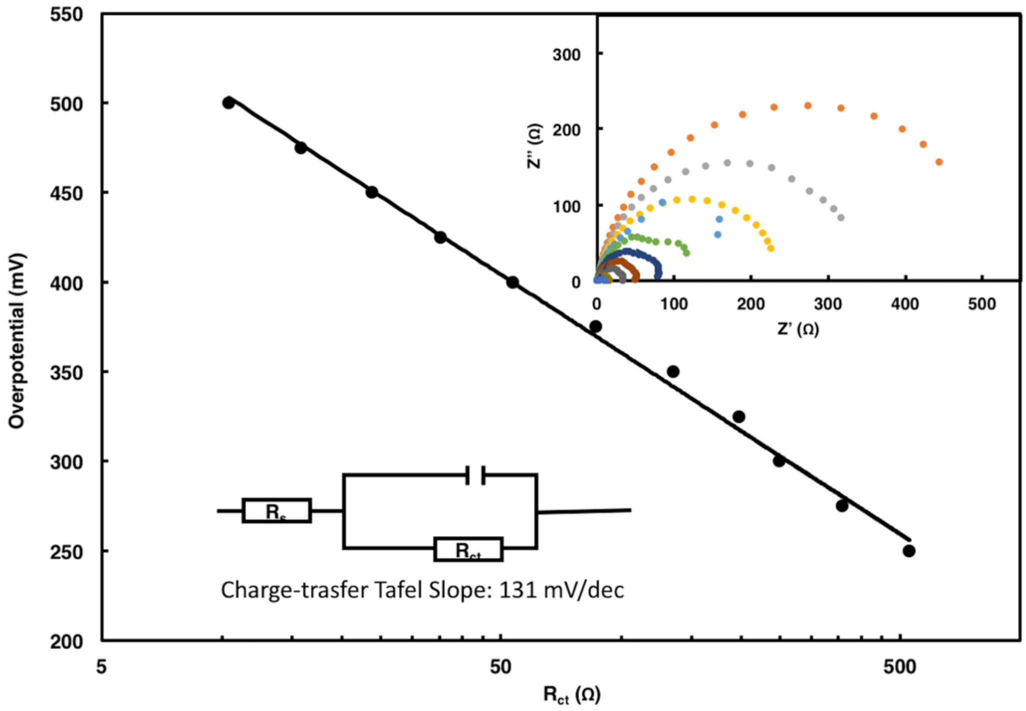

Fig. S14. Charge-transfer Tafel plots of **NiAT** from the Nyquist plots. Inset: Nyquist plots and fits of the impedance response of the vertically aligned **NiAT** sheet array catalyst under the overpotential from 250 mV to 500 mV in 25 mV increments, and the relating equivalent circuit that was used to fit the electrochemical impedance spectroscopy (EIS) data. R_s indicates the uncompensated series resistance, constant- phase element (CPE) refers to the double-layer capacitance under HER conditions, and R_{ct} represents the charge transfer resistance in the HER.

ECSA and TOF of **NiAT** (Figs. S15 and S16)

The density of the active sites on the surface can identified by the electrochemical surface area (ECSA) of the **NiAT.** ECSA was calculated by testing the electrochemical double layer capacitance (C_{dl}).

The specific capacitance can be calculated from the CV curve using the following equations:

$$C = \frac{\int ivdv}{2\mu m \Delta V} \quad (1)$$

where i and v are the current and potential in the CV test (A and V), μ is the scan rate (V/s), m is the area of electrode (cm^2), ΔV is the potential window of discharge (0.1V here, Fig. S15). Resulted C_{dl} of 2.28 mF/ cm^2 and general specific capacitance of C_s = 0.12 mF/ cm^2.

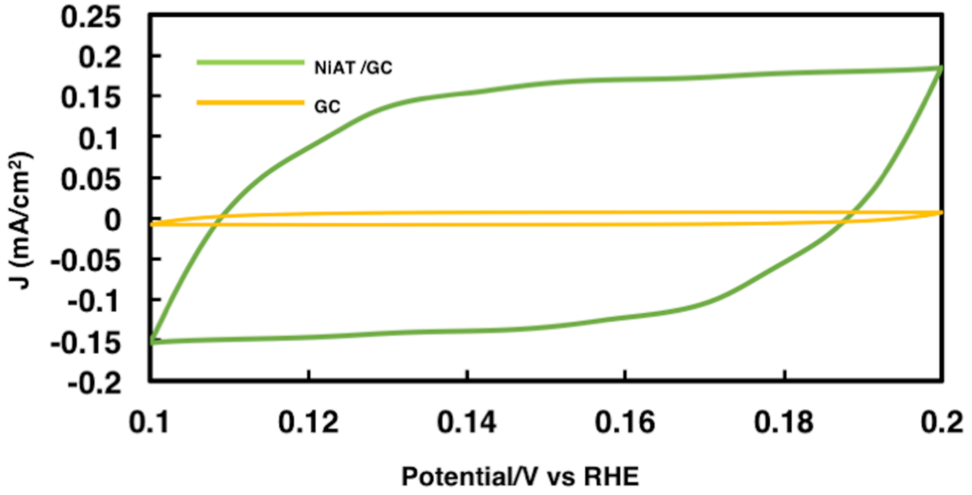

Fig. S15. Cyclic voltammograms (CV) curves of **NiAT** on glassy carbon electrode (**NiAT**/GC) and Nafion® droped glassy carbon electrode (GC) in the region of 0.1~0.2 V vs. RHE at a scan rate of 50 mV s^{-1}**.**

The ECSA of a catalyst can be calculated from the double layer capacitance (C_{dl}) and C_s according equation (2):

$$ECSA = \frac{C_{dl}}{C_s} \quad (2)$$

TOF is calculated using the current density (j) and the active site density (N) according to the following equation:

$$TOF = \frac{\text{Total number of } H_2 \text{ molecules per second}}{\text{Total number of active sites per unit area}} = \frac{j}{2qN} \qquad (3)$$

where q is the elementary charge as 1.6×10^{-19}, and 2 accounts for 2 electrons transfer per one H_2 molecule generation. The density of HER active sites in Ni atoms in **NiAT** is 7.21×10^{18} Ni atoms per cm^2 according to equation (4):

$$\#_{Ni}(0.2mg) = \frac{(0.2 / 0.07)\,\frac{mg}{cm^2}}{608.73\,\frac{g}{mol}} 6.022\times10^{23} \text{ x } 3 = 7.21 \text{ x } 10^{18} \qquad (4)$$

So the active TOF of Ni is calculated according to equation (5):

$$TOF_{Ni} = \frac{j}{2 \text{ x } 1.6 \text{ x } 10^{-19} \text{ x } \#_{Ni}(0.2mg) \text{ x } ECSA} \qquad (5)$$

The calculated TOF (Fig. S16) of **NiAT**.

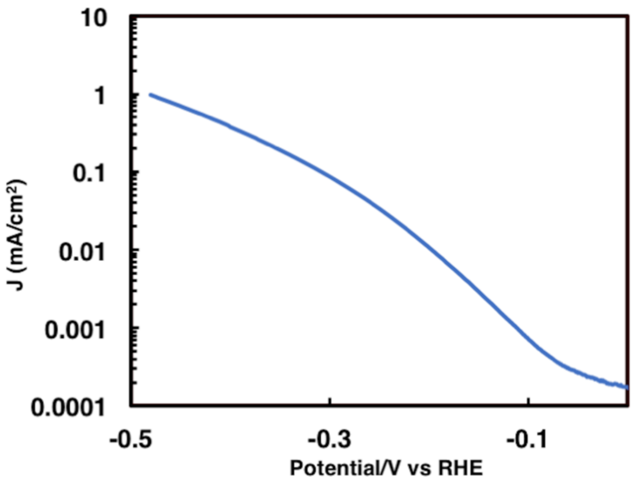

Fig. S16. Plot that displays the turnover frequency (TOF) per Ni atom **NiAT** catalysts.

Durability of **NiAT** for 500 cycles of HER (Figs. S17 and S18)

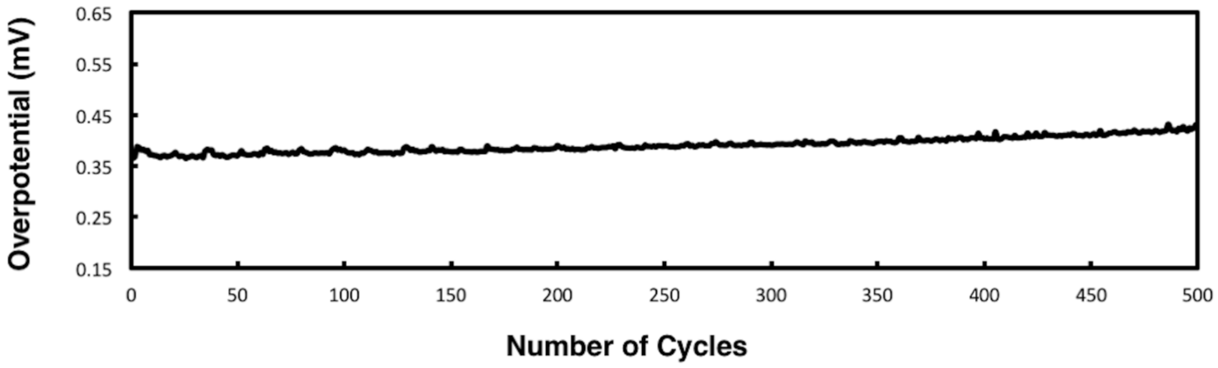

Fig. S17. Operating potential versus HER Cycles at 10 mA cm^{-2}.

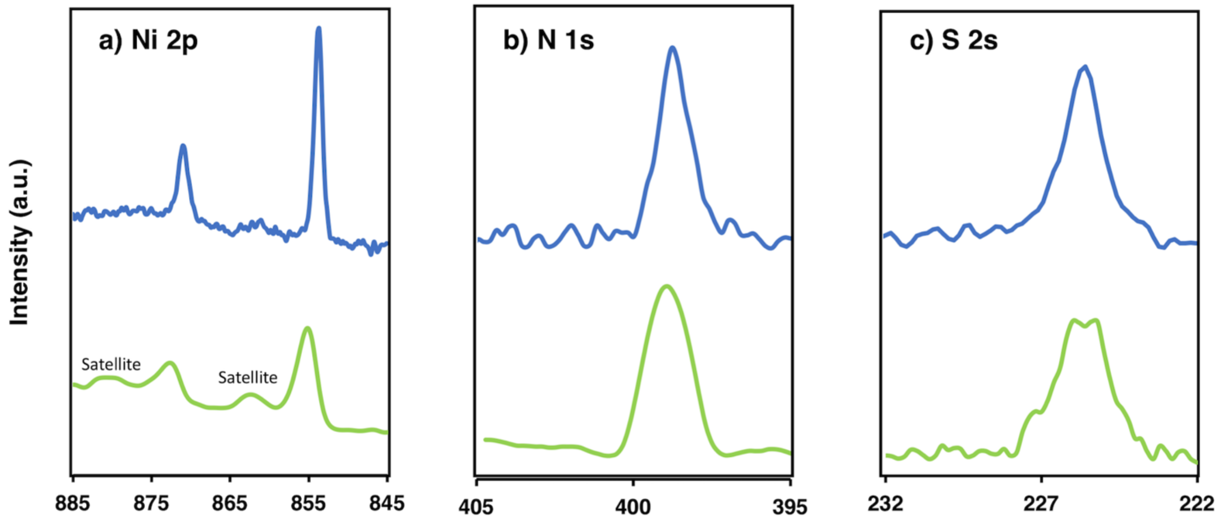

Fig. S18. **a**) Narrow-scan X-ray photoelectron spectroscopy (XPS) focusing on the N 1s region. **b**) Focusing on the S 2s region. **c**) Focusing on the Ni 2p region. Upper (red curve) before HER, lower (green curve) after HER.
